# Supplementary material for: Design and Implementation of a Time-Restricted Eating Intervention in a Randomized, Controlled Eating Study
Source: Nutrients. 2023 Apr 20;15(8):1978. doi: 10.3390/nu15081978 (PMC10144293; doi:10.3390/nu15081978)
Supplement: Supplementary file 1 [file nutrients-15-01978-s001.zip › Handout S1.pdf]

Handout S1

Guidelines for Allowed Seasonings and Flavorings

You may consume as much as you want of the following seasonings, sugar substitutes, mints, and gum. However, you are **NOT** allowed to take any sugar substitutes or sugar free mints and gum 24 hours prior to your Oral Glucose Tolerance Test (OGTT) appointment.

These do not need to be recorded on your Daily Diary.

SEASONINGS

Curry Powder
Onion Powder
Garlic Powder
Pepper, Black
Pepper, Cayenne (Red)

McCormick Perfect Pinch –
- Italian Seasoning
- Salt Free Garlic & Herb Seasoning
- Salt Free Southwest Sweet 'N Smoky Seasoning
- Salt Free Original All-Purpose Seasoning
- Salt Free Fiesta Citrus Seasoning

SUGAR SUBSTITUTES

Equal
Nutrasweet
Splenda
Stevia
Sugar Twin
Sweet' N Low

MINTS AND GUM – HAS TO BE SUGAR FREE

Altoids, sugar free
Eclipse, sugar free
Extra, sugar free
Five, sugar free
Life Saver, sugar free
Mentos, sugar free
Orbit, sugar free
Tic Tac, sugar free
Trident, sugar free

IMPORTANT - NO SALT SUBSTITUTES
