# Supplementary material for: Design and Implementation of a Time-Restricted Eating Intervention in a Randomized, Controlled Eating Study
Source: Nutrients. 2023 Apr 20;15(8):1978. doi: 10.3390/nu15081978 (PMC10144293; doi:10.3390/nu15081978)
Supplement: Supplementary file 1 [file nutrients-15-01978-s001.zip › Handout S2.pdf]

Handout S2

Guidelines for Allowed Beverages

For Time-restricted Group: No Beverages besides water after 6pm
For Usual Feeding Pattern Group: No Beverages besides water after midnight
Neither Group

IMPORTANT: You are NOT allowed to drink any artificially sweetened beverage or alcoholic beverage or Crystal Light 24 hours prior to your Oral Glucose Tolerance Test (OGTT) appointment.

Limited Allowed Beverages – Limit to 1 serving per category per day

Please remember to record your intake daily on your daily diary. This includes beverages served at your onsite meal that are not part of your TRIM Diet.

Category 1: Caffeinated Beverages

- **Brewed or Instant Coffee (regular or decaffeinated) - 8 ounces (1 cup)**

You will be provided with one liquid coffee creamer to go along with your one allowed coffee per day. You can also add some artificial sweetener (see list in the handout of Allowed Seasonings and Flavorings). Do not add anything else that is not provided to you as part of your diet.

or

- **Brewed or Instant Tea (regular or decaffeinated) - 8 ounces (1 cup)**

You can add some artificial sweetener to your one allowed tea (see list in the handout of Allowed Seasonings and Flavorings). Do not add anything else that is not provided to you as part of your diet.

Category 2: Artificially Sweetened Beverage

- **Diet Sodas and Beverages (12 ounce can or 1 ½ cup serving):**

For example,
Diet Pepsi, Diet Pepsi with Splenda(regular or decaffeinated) - 12 ounces
Diet Coke, Coca Cola Zero Sugar (regular or decaffeinated) - 12 ounces
Diet Seven-Up - 12 ounces
Diet Ginger Ale - 12 ounces

Category 3: Alcoholic Beverages

- **Wine - 5 ounces ……………………………... (1/2 cup + 2 Tablespoons)**
- **Light Beer only - 12 ounces ……………………………... (1 can or bottle)**
  (ex. Bud light, Coors Light, Miller Light)
- **Gin, Vodka, Whiskey, light Rum, 1.5 ounces ………..… (1 small jigger or 3 Tablespoons)**
  (Mix only with diet soda or a beverage from your diet for that day).
  Example:
  5.5 oz of Cranberry juice (provided on your TRIM diet) and 1.5 oz. of vodka
  or
  Diet Coke (a limited beverage) and 1.5 oz. of light rum

Free beverages - Do not need to be recorded

Crystal Light: **8 ounces (1 cup)**

Lemonade, Pink Lemonade

Bottled water & Sparkling water:

Water/Sparking water (unflavored, flavor essence only, unsweetened)
