# Supplementary material for: Design and Implementation of a Time-Restricted Eating Intervention in a Randomized, Controlled Eating Study
Source: Nutrients. 2023 Apr 20;15(8):1978. doi: 10.3390/nu15081978 (PMC10144293; doi:10.3390/nu15081978)
Supplement: Supplementary file 1 [file nutrients-15-01978-s001.zip › Handout S3.pdf]

General Dietary Information Questionnaire

TRIM ID #:

Date:

1. Do you have access to a refrigerator a) at home? Yes ☐ No ☐

   b) at work? Yes ☐ No ☐

2. Do you have access to a microwave or regular oven a) at home? Yes ☐ No ☐

   b) at work? Yes ☐ No ☐

3. Please **TICK** any of the foods below that you cannot or will not eat for religious reasons or because of allergies or severe physical discomfort (e.g., vomiting, diarrhea, severe stomach cramps):

	**VEGETABLES**				
	Potatoes		Spinach/Cooked or Raw		
	Yams		Tomato Juice		
	Broccoli/Cooked or Raw		Low Sodium V-8 Juice		
	Corn		Cabbage		
	Peas		Brussel Sprouts		
	Kale		Asparagus		
	Collards		Peppers		
	Tomato/Tomato Products				
	Lettuce				
	Carrots				
	Onions/Garlic				
	**DAIRY/EGG PRODUCTS**				
	Milk/Skim, 1%,2%		Yogurt/Plain/Fruit/Flavored		
	Cheese/ Cheddar, Provolone		Eggs/Egg Substitutes		
	Cream Cheese				
	Cottage Cheese				
	**MEAT/ POULTRY/FISH**				**VEGETARIAN/SOY PRODUCTS**
	Beef		Fish/Cod		Soy Products
	Ground Beef		Salmon		
	Pork/Ham				
	Chicken				
	Turkey				
	**NUTS**				**SEEDS**
	Pecans		Peanuts		Pumpkin Seeds
	Walnuts				Sunflower Seeds
	Almonds				
	Cashews				
	**LEGUMES**		Black Beans		
	Pinto Beans		White Beans		
	Garbanzo Beans		Kidney Beans		
	Lentils		Peanut Butter		

TRIM Study
General Dietary Information Questionnaire

TRIM ID #:

Date:

	FRUITS/JUICES				
	Oranges		Apple Juice		Raisins
	Apples		Orange Juice		Dried Apricots
	Bananas		Cranberry Juice		Dried Cranberries
	Blue Berries		Pomegranate Juice		Watermelon
	Raspberries		Kiwi		Cantaloupe
	Strawberries		Pears		
	Mango		Peaches		
	Pineapples		Cranberry Sauce		
	Applesauce		Mixed Fruit		
BREAD/GRAIN/CEREAL					
	White Bread		Corn Flakes		
	Whole Wheat Bread		Special K		
	Rice/White or Brown		Water Crackers		
	Quinoa		Pasta		
	Cheerios		Panko Crumbs		
	Oat Meal				
	Steel Cut Oats				
	Bulgar Wheat				
	Graham Crackers				
	**SNACKS/DESSERTS**		**SPICES**		
	Jello/Regular or Sugar-Free		Mint		Maple Extract
	Mints/Regular or Sugar-Free		Basil		Rum Extract
	Dove Chocolate Pieces		Vanilla Extract		Lemon Juice
			Almond Extract		Cinnamon
			Orange Peel		Nutmeg
			Oregano		Ginger
			Italian Seasoning		Pumpkin Pie Spice
			Smoked Paprika		Cloves
			Coffee Extract		Fiesta Seasoning
			Smoked Pepper		Mexican Seasoning
CONDIMENTS					
			Low Sodium Soy Sauce		Canola Oil
			Salt		Safflower Oil
			Sugar		Black Olives
			Pepper		Jelly/ Regular or Sugar-Free
			Salt Substitute		Lime Juice
			Sugar Substitute		Mayonnaise
			Mustard		Butter/margarine
			Vinegar		Olive oil

The following are a variety of questions related to your overall eating

TRIM Study
General Dietary Information Questionnaire

TRIM ID #:

Date:
environment. Your answers will help the staff determine ways they can make your participation in the study more enjoyable.

	Yes	No	
4. Do you foresee problems or difficulties in transporting, storing, refrigerating, and warming your study foods when you are away from our center?	❐	❐	
5. Do you participate in activities where food is served, such As sporting events, religious gatherings, business meetings, etc.?	❐	❐	
6. Are there special days such as holidays, birthdays, family reunions, vacations, etc., that will occur during the period you are on the study?	❐	❐	
7. If you are responsible for preparing meals in your household, will this make it difficult for you to meet study requirements?	❐	❐	NA ❐
8. Is there anyone in your household that would be affected or inconvenienced By your participation in this study?	❐	❐	

If yes, who are they and how will they be affected?

____

	Yes	No	
9. Will your employment status (e.g., job transfer) or work hours (e.g., moving to a night shift) change during the study?	❐	❐	
10. Do you, or anyone in your household, work in the food service industry (cafeteria, bakery, restaurant, etc.)?	❐	❐	
If yes, do you eat any meals or snacks at work, either as a requirement NA of your job or as a matter of convenience?	❐	❐	❐
11. Did you routinely follow a time-restricted pattern of eating in the past year? (e.g., eat all meals in ≤10 hours/day, follow intermittent fasting protocol)	❐	❐	

TRIM Study

General Dietary Information Questionnaire

TRIM ID #:

Date:

12. If you have any concerns about the study, please write them in the space provided below (use the back of this page if you need additional space).

____

____

____

____

____

13. Are there any other foods not listed above that you <u>cannot</u> or <u>will not</u> eat for any reason or because of allergies, severe physical discomfort (e.g., vomiting, diarrhea, severe stomach cramps)?

Yes ☐ No ☐

If yes, please specify

____

____

Completed by participant

Date Completed: ___________________

1st Review by (staff ID):______________

Date reviewed: _____________________

2nd Review by (staff ID): _____________

Date reviewed: _____________________
