# Supplementary material for: Design and Implementation of a Time-Restricted Eating Intervention in a Randomized, Controlled Eating Study
Source: Nutrients. 2023 Apr 20;15(8):1978. doi: 10.3390/nu15081978 (PMC10144293; doi:10.3390/nu15081978)
Supplement: Supplementary file 1 [file nutrients-15-01978-s001.zip › Handout S4.pdf]

Handout S4

TRIM

SAFE FOODS TO GO

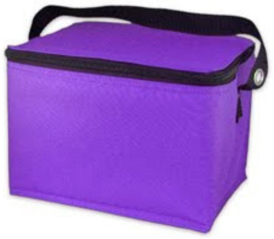

1. **What are TO GO Foods?**

TO GO foods are the meals that you pick up from your feeding study site and eat at home, work or away. You are to eat all of the food provided.

2. **What are Emergency Meals and Energy Foods.**

Emergency meals are meals that we will provide to you so that you will have a replacement meal. An emergency meal may be needed in the event that inclement weather occurs or one of your meals becomes spoiled or damaged. You are to use emergency meals only when the study staff instructs you to do so.

Energy foods are foods bars or granola that are similar in nutritional value to your study diet. They are used to adjust your calories for you to maintain your weight. At the beginning of the study, you will be given the option to eat these, should you get hungry. Later in the study you may be required to eat

them as a daily part of your TO GO meals. The study staff will let you know.

3 How do I know my TO GO foods are safe to eat?

We have carefully prepared your TO GO foods using the freshest ingredients. We follow the state's Health Department food sanitation requirements and guidelines for food preparation. The dietary managerial staff is Serve Safe Certified. <u>It is important that you, also, handle the TO GO foods safely</u>.

4. How should I store my TO GO foods to keep them safe?

In hot weather, do <u>not</u> transport TO GO Foods in the trunk of your car. Instead, you should place the foods inside your car with air conditioning.

<u>TO GO foods may contain perishable foods that require immediate refrigeration</u> (for example: meats, fish, tuna, poultry, sausage, milk, cheese, cottage cheese, yogurt, eggs, mayonnaise, salad dressing, margarine, rice, vegetables, gravy, barbeque sauce and other foods).

You should refrigerate all perishable foods at a temperature between 35 degrees F and 40 degrees F as soon as possible. TO GO foods should not remain out of the refrigeration for longer than one hour.

<u>TO GO foods may contain perishable foods that require immediate freezing</u> (such as some parts of the emergency meal). Place frozen items into the freezer within one hour of pick-up and follow instructions on the Emergency meal.

5. What if I must transport my TO GO foods for longer than one hour without Refrigeration?

You should <u>use a cooler with an ice pack</u> to transport or store TO GO foods when they will be without refrigeration for longer than one hour. Replace the ice as needed.

6. How can I tell if my TO GO foods are spoiled?

If any of your TO GO foods ever look or smell odd, your TO GO meal may be spoiled. Do NOT taste or eat the food that you think has spoiled.

7. What do I do if I suspect some of my TO GO foods may be spoiled?

Do not eat the perishable food items contained in the TO GO package if you suspect that they may be spoiled. You should immediately notify us and follow our instructions on what to do next. We may provide you a replacement food, or give you instructions to eat your emergency meal or energy cookies.

8. What should I do if I have a problem with my TO GO foods, such as an item is missing or extra when I compare the foods to my copy of the menu, or a drink has spilled?

If you discover a problem with your TO GO foods, contact us as soon a possible to receive instructions on what to do.

9. Do I need to cook my TO GO foods?

We have cooked most of the TO GO items. You may choose to reheat your meals in a microwave, toaster oven, or conventional oven.

Foods that may need to be cooked further or reheated, will be packaged in containers that are suitable for microwave heating. If you need to transfer food

To be cooked in a toaster oven or conventional oven, or if you transfer food to a plate, be sure to use a spatula and transfer all of the food. Use the instructions

Provided on the food package or included with your copy of the menu. Should you have any further questions regarding cooking instructions, please ask.

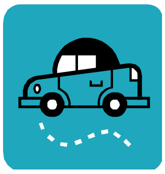

SAFELY ENJOY YOUR TO GO FOODS
