# Supplementary material for: Design and Implementation of a Time-Restricted Eating Intervention in a Randomized, Controlled Eating Study
Source: Nutrients. 2023 Apr 20;15(8):1978. doi: 10.3390/nu15081978 (PMC10144293; doi:10.3390/nu15081978)
Supplement: Supplementary file 1 [file nutrients-15-01978-s001.zip › Table S1.pdf]

Table S1. Sample TRIM Menu Items for Single Day

	TRF	UFP
**Breakfast items, g**		
Lentil kale white bean salad	93	45
Almonds, sliced	30	10
Triscuits®	10	-
Cereal, Special K®	46	46
Sugar, white, granulated	6	6
Mixed fruit, in juice, individual cup	226	-
Milk, skim, with vitamins A & D	240	240
**Lunch items, g**		
Tuna Salad	120	30
Lentil kale white bean salad	132	180
Salt, table (add to lentil kale white bean salad)	0.5	0.5
Oil, olive (add to lentil kale white bean salad)	8	-
Triscuits®	15	15
Sara Lee® Whole Wheat Bread	52	-
Jelly	14	-
Mixed fruit, in juice, individual cup	-	226
**Dinner items, g**		
TRIM Cozy Beef Stew	175	175
Salt, table (add to cozy beef stew)	0.8	0.8
Spinach, fresh, leaf	50	50
Tomatoes, cherry, red, fresh	65	65
Vinegar, rice, original seasoned	15	15
Oil, olive	-	8
Tuna Salad	-	90
Almonds, sliced	-	20
Triscuits®	-	10
Sara Lee® Whole Wheat Bread	-	52
Jelly	-	14
**Snack items, g**		
Peanuts, dry roasted, unsalted	10	10
Mandarin orange, canned, in juice	113	113
