# Supplementary material for: Design and Implementation of a Time-Restricted Eating Intervention in a Randomized, Controlled Eating Study
Source: Nutrients. 2023 Apr 20;15(8):1978. doi: 10.3390/nu15081978 (PMC10144293; doi:10.3390/nu15081978)
Supplement: Supplementary file 1 [file nutrients-15-01978-s001.zip › Table S2.pdf]

Table S2. Standardized Procedure for Obtaining Recipe Cooked Factors

Step	Procedure
1.	Record the weight of the empty pan before adding any raw ingredients (**pan weight**).
2.	When all the raw ingredients have been weighed and added to the pan, record the weight (**raw weight**).
3.	After the cooking and cooling procedures are followed exactly and food cooled down to 40 °F, record the weight of the cooked recipe in the pan (**cooked weight**).
4.	Cooked Factor = (cooked weight – pan weight) / (raw weight – pan weight) x 100.<br>This number is expressed as a percent which is then multiplied by the calculated food amount.
5.	Repeat steps 1 to 3 for two more times, add the three cooked factors and divide by 3 for the averaged cooked factor.
Example	If menu lists 100 grams of Chili (based on raw weight of ingredients) as the menu item, and if the cooked factor is estimated to be 83% based on Steps 1-5 above, then, 83 grams of Chili should be specified on the production sheet, rather than 100 grams, to account for weight loss during cooking.
