# Supplementary material for: Design and Implementation of a Time-Restricted Eating Intervention in a Randomized, Controlled Eating Study
Source: Nutrients. 2023 Apr 20;15(8):1978. doi: 10.3390/nu15081978 (PMC10144293; doi:10.3390/nu15081978)
Supplement: Supplementary file 1 [file nutrients-15-01978-s001.zip › Table S3.pdf]

Table S3. Physical Activity Factors, Categories, and Definitions

**Activity factors**	**Physical Activity Category**	**Definition**[1]
1.3	Sedentary	Activity only associated with typical day-to-day life.
1.4	Low	Not meeting any of the criteria for either of the moderate or high category.
1.5	Moderate	Doing some activity. It is a level of activity equivalent to half an hour of at least moderate-intensity physical activity on most days.
1.6	High	Requiring higher levels of participation. It is a level of activity equivalent to at least one hour of moderate-intensity physical per day or at least half an hour of vigorous-intensity activity per day.

1 Committee on Diet and Health, National Research Council. Diet and health: implications for reducing chronic disease risk. ISBN: 0-309-58831-6 (1989) https://www.ncbi.nlm.nih.gov/books/NBK218743/pdf/Bookshelf_NBK218743.pdf, Accessed February 14, 2021.
