# Supplementary material for: Design and Implementation of a Time-Restricted Eating Intervention in a Randomized, Controlled Eating Study
Source: Nutrients. 2023 Apr 20;15(8):1978. doi: 10.3390/nu15081978 (PMC10144293; doi:10.3390/nu15081978)
Supplement: Supplementary file 1 [file nutrients-15-01978-s001.zip › Table S4.pdf]

Table S4. Average Percentage of Daily Calories by Meal in the Time-Restricted Feeding Arm

	1600 kcal daily			2000 kcal daily			2500 kcal daily			3000 kcal daily		
**Meal**	**Target**	**Average**	**SD**	**Target**	**Average**	**SD**	**Target**	**Average**	**SD**	**Target**	**Average**	**SD**
Breakfast	40	39.6	0.3	40	39.9	0.5	40	39.9	0.7	40	39.9	0.9
Lunch	40	39.5	1.1	40	39.6	0.9	40	39.8	0.5	40	39.8	0.9
Dinner	15	15.8	0.9	15	15.2	0.7	15	15.2	0.8	15	15.4	1.1
Snack	5	5.1	0.4	5	5.4	0.3	5	5.0	0.4	5	5.0	0.3

Abbreviations: SD, standard deviation.

Note: All results are provided in %. No participant had a 3500 kcal diet in the Time-Restricted Feeding Arm.
