# Supplementary material for: Design and Implementation of a Time-Restricted Eating Intervention in a Randomized, Controlled Eating Study
Source: Nutrients. 2023 Apr 20;15(8):1978. doi: 10.3390/nu15081978 (PMC10144293; doi:10.3390/nu15081978)
Supplement: Supplementary file 1 [file nutrients-15-01978-s001.zip › Table S5.pdf]

Table S5. Average Percentage of Daily Calories by Meal in the Usual Feeding Pattern Arm

	1600 kcal daily			2000 kcal daily			2500 kcal daily			3000 kcal daily			3500 kcal daily		
**Meal**	**Target**	**Average**	**SD**	**Target**	**Average**	**SD**	**Target**	**Average**	**SD**	**Target**	**Average**	**SD**	**Target**	**Average**	**SD**
Breakfast	20	20.1	0.6	20	19.7	0.5	20	20.1	0.6	20	19.5	0.7	20	20.1	0.2
Lunch	25	24.9	0.3	25	25.6	1.5	25	25.0	0.6	25	25.3	0.4	25	25.0	0.2
Dinner	50	50.1	1.0	50	49.3	1.6	50	49.5	0.9	50	50.2	0.6	50	49.9	0.2
Snack	5	4.9	0.6	5	5.4	0.3	5	5.4	0.5	5	5.0	0.3	5	5.0	0.2

Abbreviations: SD, standard deviation
Note: All results are provided in %.
